# Coma in Diabetes Insipidus

**Published:** 1904-09-24

**Authors:** 


### COMA IN DIABETES INSIPIDUS.

Everyone is familiar with the dreaded complication of coma in cases of diabetes mellitus. Its frequently rapid development and almost invariably fatal termination, make it one of the most unhappy possibilities of this disease. Professor Alfred Carter[1] has recently reported an instance in which fatal coma supervened in a case of diabetes insipidus. The patient was a girl of eight years. Thirst and frequency of micturition were first noted on June 25, and signs of drowsiness came on as early as July 10; the patient died on July 13. There was no history of injury, excitement, or exposure to wet, all of which conditions have been recorded as leading to the development of acute polyuria. Hence the case is unusual, not only in respect to its fatal complication, but also in reference to its acute onset and course. There are many circumstances which point to a relationship between diabetes mellitus and diabetes insipidus. Professor Carter's case shows that among these the possibility of a fatal coma must be included. Trousseau used to teach that children with diabetes insipidus had not infrequently glycosuric parents, and a tubercular family history is certainly not uncommon in both diseases. In a discussion at the Association of American Physicians,[2] Dr. Jacobi said that diabetes insipidus in children was not a rare disease; he had found it in association with chorea and other convulsive disorders, and in cases of hereditary syphilis. For treatment he relied mainly on potassium iodide, but also found ergot of value. In Professor Carter's case the injection of saline fluid was tried, but without avail.

[1] Lancet, August 27, 1904. [2] Ibid., June 4, 1904.

